# Knowledge, risk perception and household practices related to indoor chemical pollution in the United Arab Emirates

**DOI:** 10.3389/fpubh.2026.1926277

**Published:** 2026-10-21

**Authors:** Wissam Ghach, Rina Salhab, Massa Nakad, Mira Qoli, Nisreen Alwan, Emran Alotaibi

**Affiliations:** 1 Department of Public Health, Canadian University Dubai, Dubai, United Arab Emirates; 2 Department of Environmental and Public Health, Abu Dhabi University, Abu Dhabi, United Arab Emirates

**Keywords:** household practices, indoor chemical pollution, knowledge, risk perception, self-reported symptoms, United Arab Emirates

## Abstract

**Introduction:**

Indoor chemical pollution is a residential exposure concern shaped by building materials, ventilation and everyday household practices, yet evidence remains limited in hot-arid, highly air-conditioned settings.

**Materials and methods:**

This cross-sectional questionnaire-based study used a non-probability convenience sample of 485 adult UAE residents. Knowledge, risk-aware attitudes, protective and source-generating practices, a formative safe-practice index, and nonspecific self-reported symptoms were analyzed. Continuous knowledge, attitude, and safe-practice scores were emphasized in primary analyses; study-defined thresholds were retained only for secondary descriptive or sensitivity analyses.

**Results:**

The mean knowledge score was 4.32/9 (SD 2.08) and the mean risk-aware-attitude score was 5.75/11 (SD 2.24). In adjusted models, each standard-deviation increase in knowledge was associated with a 0.66-point higher attitude score (95% CI 0.47–0.86), and each standard-deviation increase in risk-aware attitude was associated with a 2.67-point higher safe-practice index (95% CI 1.63–3.71). Source-generating practice was associated with a higher symptom count (IRR 1.16 per SD, 95% CI 1.02–1.32), whereas its association with any symptom was not statistically supported (OR 1.11, 95% CI 0.89–1.38).

**Conclusion:**

Within this demographically skewed convenience sample, knowledge, attitudes and household practices showed cross-sectional associations, and source-generating practices were modestly associated with greater nonspecific self-reported symptom burden. The data do not establish a causal knowledge-attitude-practice pathway or that indoor chemicals caused reported symptoms. Representative sampling and objective exposure and health measurements are needed before population-level or causal conclusions can be drawn.

## 1 Introduction

Residential indoor air is a major exposure microenvironment because time-activity patterns concentrate daily inhalation, dermal, and mucosal contact within homes, classrooms, offices and vehicles. Human exposure is therefore not determined only by outdoor concentrations, but by the combination of indoor sources, ventilation, filtration, deposition, resuspension and indoor chemical transformation (1, 2). Home environment is particularly important because occupants can introduce or intensify chemical sources through cooking, cleaning, heating, smoking, fragrance and pest-control practices, while modern air-tight or mechanically cooled buildings may reduce dilution when outdoor air exchange is limited. Indoor chemistry further complicates exposure assessment because emitted volatile organic compounds, oxidants and surface residues can react indoors to produce secondary pollutants not directly released from the original source (3, 4).

A growing body of peer-reviewed research shows that common residential activities can generate health-relevant indoor pollutants (5, 32). Gas cooking is a recognized source of nitrogen dioxide and other combustion-related pollutants, and pooled epidemiological evidence links indoor nitrogen dioxide and gas cooking with asthma and wheeze, especially in children (6). Cleaning products and air fresheners can release terpenes, glycol ethers and other volatile chemicals, and reactions with ozone may produce secondary formaldehyde, ultrafine particles and oxygenated products that increase exposure complexity (4, 7). Formaldehyde remains a priority indoor pollutant because of emissions from pressed-wood products, coatings, furnishings and chemical reactions, as well as its irritant properties and carcinogenic relevance (8, 9). Tobacco smoke also extends beyond active smoking episodes: nicotine and other residues can sorb to surfaces and dust, persist as third-hand smoke and react with indoor nitrous acid to form tobacco-specific nitrosamines (10, 11).

These sources are not equally visible to householders. Combustion odor, smoke and dust may be noticed quickly, whereas formaldehyde, ozone-initiated chemistry and residue-mediated third-hand smoke are often invisible and chemically abstract. This perceptual asymmetry matters for prevention because risk control in homes depends on behavior as well as technology. Risk communication studies and the Health Belief Model suggest that perceived susceptibility, severity, benefits and practical barriers can shape preventive action even when factual knowledge exists (12). In indoor air research, this creates a knowledge-attitude-practice (KAP) problem: knowing that indoor air can be polluted may not translate into opening windows at appropriate times, maintaining extraction systems, avoiding unnecessary fragranced products, using low-emission materials or restricting smoking indoors (12, 13).

The Gulf Cooperation Council (GCC) region presents a distinctive indoor-air context. Hot climate, dust events, high reliance on air-conditioning, rapid urban development, multi-residential buildings and culturally embedded indoor scenting practices can create exposure profiles that differ from temperate settings where much of the indoor-air literature originated. Reviews of GCC indoor air quality identify important gaps in pollutant measurement, exposure assessment, source apportionment and health linkage (14). A recent United Arab Emirates (UAE) research strategy further emphasized the need for systematic indoor-source characterization, behavioral evidence, vulnerable-population assessment and national-scale monitoring that can inform standards and interventions under local climatic and building conditions (15). These reviews indicate that imported assumptions about residential exposure may be insufficient for the UAE without local behavioral and epidemiological data.

UAE-specific empirical evidence has begun to define the problem but remains incomplete. A large household investigation of 628 UAE homes reported that daily incense use was associated with symptoms such as headache, difficulty concentrating and forgetfulness, indicating that culturally common indoor sources may have measurable health correlates (16). Laboratory and exposure-oriented work have also shown that Arabian incense smoke can contain particles and organic compounds of toxicological concern (17, 18). However, existing UAE studies have tended to focus either on pollutant measurement and source hazards or on awareness in specific student groups. Recent student-centered studies documented limited indoor-air awareness and perception among health-professional and university populations in the UAE but did not provide a community-level behavioral model connecting chemical knowledge, risk attitudes, multiple household practices and symptom burden in the same analytic framework (19, 20).

Sick Building Syndrome (SBS), or building-related symptoms, provides a useful symptom-based framework for interpreting nonspecific indoor-environment health complaints. Classically, SBS refers to symptoms such as headache, fatigue, dizziness, eye and throat irritation, respiratory discomfort and skin irritation that are associated with time spent indoors but are not readily explained by a single diagnosed disease (21, 22, 33). Although SBS was historically studied mainly in offices and institutional buildings, more recent indoor-environment research has shifted toward broader indoor environmental quality, healthy-building performance, ventilation, pollutant exposure and occupant-reported health outcomes (2, 23–25). This broader interpretation is relevant to homes in hot-arid, highly air-conditioned settings such as the UAE, where prolonged indoor occupancy, mechanical cooling, limited window-opening behavior, fragranced products, combustion sources, tobacco smoke residues, cleaning agents and building-material emissions may interact with ventilation and filtration practices. Therefore, SBS is not treated in this study as a clinical diagnosis, but as an indoor-environment symptom framework that helps interpret self-reported symptoms alongside household chemical sources and preventive practices.

The international knowledge-attitude-practice literature also leaves a localization gap. A structured household chemical-pollution survey in Italy demonstrated that determinants of knowledge and attitudes can be assessed with source-specific questionnaire items, yet transferability to the UAE is uncertain because climate control, household routines, scenting practices, housing stock and risk communication environments differ substantially (13). The resulting evidence gap is not simply whether residents have heard of indoor air quality; it is whether residents distinguish evidence-supported hazards from misconceptions, whether risk-aware attitudes track-reduction behavior, and whether patterns of household practice correspond to symptom reporting in a UAE sample.

The present study addresses that gap through a cross-sectional questionnaire-based study of a convenience sample of 485 adult UAE residents. Data were collected specifically for this study. The objectives were to describe the sample; quantify confirmed knowledge alignment and risk-aware attitudes; derive protective, source-generating and formative safe-practice indices from household behaviors; and examine cross-sectional associations among knowledge, attitudes, practices and nonspecific self-reported symptoms after demographic adjustment. The contribution is a hypothesis-generating behavioral and symptom-oriented layer of UAE indoor-air evidence that complements pollutant-measurement studies and can inform the design of future representative research and source-specific risk communication.

## 2 Materials and methods

### 2.1 Study design and reporting approach

This cross-sectional questionnaire-based study examined knowledge, risk perception, household practices and nonspecific self-reported symptoms related to indoor chemical pollution among adult UAE residents. Data were collected specifically for the present study between October 2025 and March 2026. Because each respondent contributed data at one time point, the findings describe cross-sectional associations rather than temporal, mediating or causal relationships; in particular, reported symptoms cannot be attributed to indoor chemicals. Reporting followed the general principles of the Strengthening the Reporting of Observational Studies in Epidemiology (STROBE) statement for cross-sectional studies (34).

### 2.2 Recruitment, eligibility and data collection

Participants were recruited using a non-probability convenience-sampling approach between October 2025 and March 2026. The questionnaire was administered through Google Forms, circulated through university networks, and additionally completed during in-person recruitment using tablets. The same questionnaire was available in English and Arabic. Eligibility required residence in the United Arab Emirates and age 18 years or older; UAE citizenship and a minimum duration of UAE residence were not required. Participation was voluntary and no financial or other incentives were provided. Google Forms required sign-in and was configured to limit submission to one response per signed-in Google account, reducing duplicate participation by the same individual. Participation was not restricted at household level, so more than one eligible adult from the same household could potentially participate. Because recruitment did not use a defined population sampling frame, a recruitment denominator and formal response rate could not be calculated. All descriptive estimates are therefore unweighted sample estimates and are not national population estimates.

### 2.3 Survey instrument development, adaptation and main domains

The questionnaire was adapted from the knowledge-attitude-practice instrument developed by Montuori et al. (13) for household chemical indoor pollution. In that source study, the questionnaire was developed by a multidisciplinary group comprising physicians, chemists and biologists and was pilot-tested with 10 individuals to assess item comprehension. The UAE questionnaire retained the same broad KAP structure but introduced context-specific adaptations, including an incense attitude item, a five-level practice-frequency scale, simplification of the heater item to gas-heater use, consolidation of home-smoking items, and a study-specific multi-select symptom question. The complete English and Arabic questionnaire, item provenance, response options and scoring rules are provided in Supplementary Table S1 and Appendix S1. A separate UAE-specific cognitive pilot, formal translation/back-translation procedure and external criterion validation were not documented; therefore, the resulting scores are treated as study-specific measures rather than independently validated instruments for the UAE population.

The administered questionnaire contained demographic questions, 12 knowledge statements, 12 attitude statements, 12 household-practice questions and one multi-select symptom question. Nine of the 12 knowledge statements were included in the confirmed-knowledge score; three context-dependent, numerical or jurisdiction-specific statements were retained descriptively but excluded from that score. Eleven of the 12 attitude statements were included in the risk-aware-attitude score; the statement that it is nice to have plants in the house was treated as an aesthetic preference and excluded. Eleven practice items contributed to the practice indices, while the plant-decoration item was reported descriptively only. The symptom question allowed multiple responses and was analyzed as symptom count and any symptom. Item-level distributions for all administered knowledge, attitude and practice items are reported in Supplementary Table S2.

### 2.4 Data cleaning and recoding

Age responses were reviewed for consistency before analysis. Numeric ages within plausible limits were retained, and year-of-birth or date-like entries were converted using the response year when unambiguous. Apparent values below the adult eligibility threshold were verified with the respective respondents: eight entries recorded as 17 years were confirmed as age 18, and one entry recorded as 14 years was confirmed as age 24. These were data-entry errors and were corrected before final analysis; consequently, no participant younger than 18 years was included. Four other age responses could not be reliably resolved and were coded as missing. Cleaned age was grouped as 18–24, 25–34, 35–44 and ≥45 years.

For analysis, emirate of residence was grouped as Dubai, Abu Dhabi and Other Emirates; education was grouped as high school or less, bachelor degree and postgraduate; and smoking was coded as current smoker or non-smoker. Missing categories were shown in descriptive summaries where relevant. Adjusted models used complete-case analysis. The only model-variable missingness was the four unresolved age values; therefore, all adjusted models included 481 respondents, and no other model variables caused additional exclusions (Supplementary Figure S1).

### 2.5 Knowledge score

The confirmed-knowledge alignment score used nine items with a stable evidence-aligned direction for the present analysis. The evidence-aligned responses were: disagreement that household indoor chemical pollution is lower than outdoors; agreement that gas stoves contribute to household pollution; disagreement that plants at night release substances dangerous to health; agreement with the second-hand-smoke and third-hand-smoke definitions; disagreement that third-hand smoke is less toxic than second-hand smoke; agreement that formaldehyde is a household chemical pollutant and a carcinogen; and agreement with the stated SBS definition. Each evidence-aligned response received 1 point. An uncertain or evidence-misaligned response received 0 points in this confirmed-knowledge count, but uncertainty was retained as a separate item-level category and was not interpreted as equivalent to an affirmative false belief. Three administered knowledge statements, ventilation causing more than 50% of household chemical pollution, carbon monoxide being the main household pollutant and the existence of laws governing domestic pollution, were reported descriptively but excluded from the score because their numerical, contextual or jurisdiction-specific wording did not support a stable universal evidence-aligned classification for this analysis.

The continuous confirmed-knowledge score ranged from 0 to 9 and was used in primary descriptive, correlational and multivariable analyses. The previously used ≥6/9 category is not a validated definition of adequate knowledge. It is retained only as a study-defined secondary descriptive category for comparability with earlier analyses. Sensitivity descriptions at ≥5/9 and ≥7/9 are provided in Supplementary Table S4.

### 2.6 Risk-aware attitude score

Risk-aware attitudes were scored using 11 of the 12 administered attitude statements. Agreement was classified as risk-aware for opening windows, opening windows while air conditioning is operating and winter ventilation. Disagreement was classified as risk-aware for statements normalizing house cleaning as a waste of time, equivalence of all cleaning products, spray deodorant use, candle burning, incense burning, gas-stove preference relative to induction, fireplaces as household decoration and smoking on the sofa. The plant-aesthetic statement was excluded because it does not have a clear indoor-chemical-risk direction. These classifications operationalize study-specific risk orientation; they are not validated clinical or regulatory standards and do not imply that any single ventilation behavior is appropriate under all outdoor environmental conditions.

The continuous risk-aware-attitude score ranged from 0 to 11 and was used as the primary attitude measure. The previously used ≥8/11 category is retained only as a study-defined secondary descriptive and sensitivity outcome and is not presented as an externally validated threshold. Sensitivity logistic models using thresholds of ≥7/11, ≥8/11 and ≥9/11 are reported in Supplementary Table S4.

### 2.7 Household practice indices

Household practice responses were coded from 0 to 4 (Never, Rarely, Sometimes, Often, Always). Six protective items were prespecified for the protective-practice index: ventilating the home, using air purifiers, operating the kitchen hood, using HVAC filters, periodically checking HVAC/ventilation systems and washing curtains or carpets. Five source-generating items were prespecified for the source-generating-practice index: gas-stove use, gas-heater use, insecticide use, air-freshener use, and smoking inside the home.

The protective-practice index was the mean frequency of the six protective behaviors rescaled to 0–100, and the source-generating-practice index was the mean frequency of the five source-generating behaviors rescaled to 0–100. Higher protective scores represent more frequent reported protective behaviors; higher source-generating scores represent more frequent reported source-generating behaviors. These component indices were analyzed separately in symptom models and sensitivity analyses.

The formative safe-practice index combined the six protective items with reverse-coded values for the five source-generating items: 100 × [ΣPᵢ + Σ(4 − Sj)]/[4 × (6 + 5)]. Equal item weighting was selected as a transparent, non-data-driven aggregation rule because validated UAE-specific exposure or health-effect weights were unavailable; equal weighting does not imply that the component behaviors have equal exposure or health consequences. The continuous index was used as the primary combined practice measure. A median split at the observed median of 61.36 (reported as 61.4) was retained only as a secondary exploratory category and is termed an “above-median safe-practice score,” not “high” or objectively safe practice. An alternative domain-balanced index giving 50% weight to the protective domain and 50% to the reverse-coded source-generating domain was evaluated in sensitivity analysis.

The plant-decoration practice item was reported descriptively but excluded from the practice indices because it is neither a direct protective ventilation/maintenance behavior nor a source-generating behavior comparable to combustion, pesticides, fragranced products or indoor smoking. Its exclusion and the role of every practice item are documented in Supplementary Table S1.

### 2.8 Symptom outcomes

Self-reported symptoms were analyzed from the multi-select symptom question. “None of the above” did not contribute to symptom count. The questionnaire did not establish symptom onset, duration, clinical diagnosis, indoor location, temporal relation to a household activity, measured pollutant exposure, or alternative causes. The first outcome was symptom count, defined as the number of listed symptoms selected (observed range 0–12). The second was any symptom, coded 1 when at least one listed symptom was selected and 0 otherwise.

Because these outcomes are nonspecific self-reports, they represent symptom reporting or symptom burden only. They are not indicators of confirmed poisoning, clinical diagnoses, Sick Building Syndrome diagnoses, or verified effects of indoor chemical exposure. The Arabic version of the symptom prompt used poisoning-related wording, whereas the English prompt asked about regularly experienced symptoms; this language/framing difference and the absence of formal translation/back-translation are acknowledged as measurement limitations.

### 2.9 Measurement properties and construct-validity assessment

Internal consistency was calculated for transparency. Cronbach’s alpha was 0.631 for the knowledge score, 0.650 for the attitude score and 0.258 for the formative safe-practice index. The very low alpha for the safe-practice index confirms that its component behaviors do not covary as a unidimensional reflective scale. Because the index intentionally combines noninterchangeable behaviors, alpha is not treated as a stand-alone validity criterion for this formative construct (26, 27). The low value nevertheless reinforces that the combined index should be interpreted cautiously and alongside its protective and source-generating components.

Measurement evaluation therefore emphasized transparent content mapping and sensitivity analyses rather than claiming formal psychometric validation. All item roles and scoring directions are documented in Supplementary Table S1; item-level distributions are provided in Supplementary Table S2; protective and source-generating components are reported separately; and the alternative domain-balanced safe-practice index was strongly correlated with the original index (r = 0.992) and yielded the same directional knowledge and attitude associations. These checks do not replace external validation against observed household behavior, building characteristics or pollutant measurements.

### 2.10 Statistical analysis

Descriptive statistics summarized the analytic sample and study measures. Categorical variables were reported as counts and unweighted percentages. Continuous and ordinal-derived scores were summarized using means, standard deviations, medians, interquartile ranges and observed ranges. Selected proportions were accompanied by Wilson 95% confidence intervals; because the sample was non-probability and unweighted, these intervals describe statistical precision under the analytic assumptions and are not design-based UAE population intervals.

Item-level knowledge and attitude responses were summarized descriptively as aligned/risk-aware, uncertain or misaligned, while all practice items were summarized across Never, Rarely, Sometimes, Often and Always. No inferential *p*-values were used to declare item-level “discoveries.” Spearman rank correlations among continuous knowledge, attitude, protective-practice, source-generating-practice, safe-practice and symptom-count measures were treated as exploratory. Benjamini–Hochberg false-discovery-rate (FDR) adjusted q-values were calculated across the 15 pairwise correlations as a multiplicity sensitivity analysis (Supplementary Table S5).

Primary adjusted analyses emphasized continuous scores. Ordinary least-squares regression with HC3 heteroskedasticity-robust standard errors modeled the continuous risk-aware-attitude score as a function of standardized knowledge and modeled the continuous safe-practice index as a function of standardized knowledge and attitude. Logistic regression modeled any symptom. Negative-binomial regression modeled symptom count. Continuous composite predictors were standardized so that estimates represent a one-standard-deviation increase. All models adjusted for gender, age group, education, smoking status and emirate of residence. Reference categories were female, age 18–24 years, bachelor degree, non-smoker and Dubai.

Negative-binomial regression was selected for symptom count because variance substantially exceeded the mean in the complete-case sample (mean 1.61, variance 3.80) and an ordinary Poisson model showed overdispersion (Pearson *χ*^2^/df = 2.12; deviance/df = 2.15). The estimated negative-binomial dispersion parameter was α = 0.79 (95% CI 0.57–1.01). Observed zero counts comprised 39.9% of complete cases, compared with 37.5% predicted by the negative-binomial model. Zero inflation was additionally examined using a zero-inflated negative-binomial sensitivity model; it produced a lower AIC (1644.1 versus 1654.8 for the negative-binomial model) but a similar source-generating-practice association. The simpler negative-binomial model was retained as primary because no distinct structural-zero subgroup had been prespecified and zero symptoms are a plausible observed count.

Complete-case analysis was used for adjusted models, yielding n = 481 because four age values could not be resolved. No other model variables caused additional exclusions. Full coefficient tables with all covariates and reference categories are provided in Supplementary Table S3. Effect sizes and 95% confidence intervals are emphasized throughout; *p*-values are reported as supporting inferential information rather than definitive evidence in isolation.

Analyses were rerun reproducibly from the study dataset using Python 3.13.5 with pandas 2.2.3, NumPy 2.3.5, SciPy 1.17.0, statsmodels 0.14.6 and Matplotlib 3.10.8. During revision, ChatGPT (OpenAI; GPT-5.6 Sol; https://openai.com/) was used to assist with language editing, organization of reviewer responses, and drafting/refinement of Python code used to verify statistical analyses and figure generation. All computations were executed using the listed statistical libraries on the study data, outputs were checked against the source dataset, and responsibility for the scientific content and interpretation remains with the authors.

### 2.11 Sensitivity and multiplicity analyses

Sensitivity analyses assessed dependence on study-defined thresholds, practice-index construction and count-model specification. For the attitude outcome, the adjusted association of standardized knowledge was evaluated using ≥7/11, ≥8/11 and ≥9/11 thresholds. For the safe-practice construct, the original equal-item-weight continuous index was compared with a domain-balanced alternative that assigned 50% weight to the protective domain and 50% to the reverse-coded source-generating domain, and the protective and source-generating components were modeled separately. The former median-defined binary safe-practice outcome was retained only as a secondary analysis and renamed “above-median safe-practice score.”

For symptom count, an HC3 robust Poisson model and a zero-inflated negative-binomial model were used as sensitivity analyses. Numerical estimates, 95% confidence intervals, *p*-values and sample sizes are provided in Supplementary Table S4. For exploratory Spearman correlations, Benjamini–Hochberg false-discovery-rate (FDR) q-values are reported in Supplementary Table S5.

### 2.12 Ethical considerations

The Institutional Review Board (IRB) at Canadian University Dubai approved the study, which was conducted in accordance with the Declaration of Helsinki. The first page of the Google Forms questionnaire contained written informed-consent information describing the study purpose, potential risks and benefits, confidentiality protections and voluntary participation, and submission indicated consent to participate. Eligibility was restricted to adults aged 18 years or older. Apparent sub-18 age entries identified during data cleaning were verified directly with the respondents and confirmed to be data-entry errors (eight 17-year entries were age 18; one 14-year entry was age 24). Thus, all 485 respondents in the analytic sample were adults and parental or guardian consent was not applicable.

## 3 Results

### 3.1 Study population and analytic data structure

The analytic sample contained 485 adult respondents. After correction of verified age-entry errors, 481 respondents had usable age information; four age values remained unresolved. Median age was 30 years (IQR 20–42), with 43.5% aged 18–24 years and 18.6% aged ≥45 years. Females comprised 75.7% of the sample, Dubai residents 67.6% and Abu Dhabi residents 23.3%. Bachelor-degree holders comprised 63.5% and postgraduate respondents 24.7%. Current smoking was reported by 26.4%, children in the household by 41.2%, and prior hospitalization attributed by respondents to chemical poisoning/exposure by 2.1% (Table 1). All descriptive estimates and adjusted associations are sample-specific and should not be interpreted as UAE population estimates. Supplementary Figure S1 summarizes participant and analytic-sample flow.

**Table 1:** Demographic and household characteristics of the analytic convenience sample (*n* = 485).

Variable	Level	*n*	%
Gender	Female	367	75.7
	Male	118	24.3
Age group	18–24	211	43.5
	25–34	51	10.5
	35–44	129	26.6
	≥45	90	18.6
	Missing	4	0.8
Emirate	Dubai	328	67.6
	Abu Dhabi	113	23.3
	Other Emirates	44	9.1
Education	High school or less	57	11.8
	Bachelor	308	63.5
	Postgraduate	120	24.7
Smoking habit	Yes	128	26.4
	No	357	73.6
Children in household	Yes	200	41.2
	No	283	58.4
	Missing	2	0.4
Prior hospitalization due to chemical poisoning/exposure	Yes	10	2.1
	No	472	97.3
	Missing	3	0.6

Percentages are unweighted sample estimates. Age percentages use the full analytic denominator and include four missing age values. All respondents were adults aged ≥18 years after verification of apparent data-entry errors.

Figure 1 presents selected analytic-sample characteristics only; outcome prevalence bars were removed to avoid mixing sample descriptors with study outcomes. Continuous knowledge, attitude and practice-score distributions are summarized in Table 2, while item-level distributions are presented in Figures 2, 3 and Supplementary Table S2.

**Figure 1:**
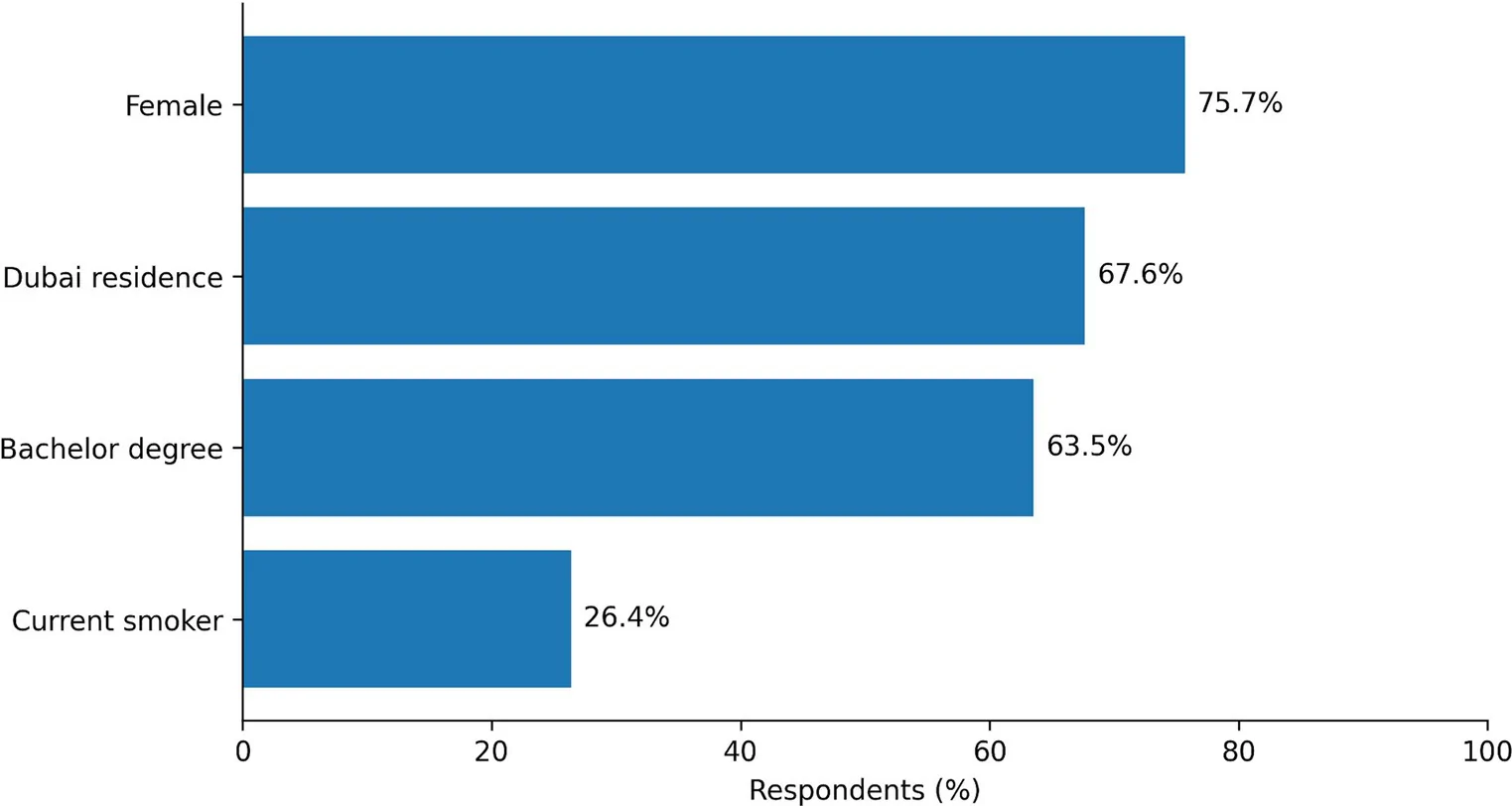
Selected analytic-sample characteristics (*n* = 485). Bars show unweighted percentages for female sex, Dubai residence, bachelor-degree education and current smoking. Outcome prevalence estimates were removed from this figure to separate sample description from study outcomes. These values characterize the analytic convenience sample and are not UAE population estimates.

**Table 2:** Continuous study measures and secondary descriptive categories in the analytic sample (*n* = 485).

Statistic type	Measure	Mean (SD)/n/N	Median (IQR)/% (95% CI)	Range
A. Continuous measures	Knowledge alignment (0–9)	4.32 (2.08)	4.0 (3.0–6.0)	0–9
	Risk-aware attitude (0–11)	5.75 (2.24)	6.0 (4.0–7.0)	0–11
	Protective-practice index (0–100)	65.21 (18.53)	66.7 (54.2–79.2)	0–100
	Source-generating-practice index (0–100)	42.04 (21.79)	40.0 (25.0–55.0)	0–100
	Safe-practice index (0–100)	61.92 (11.59)	61.4 (54.5–70.5)	25–100
	Symptom count (0–12)	1.61 (1.94)	1.0 (0.0–3.0)	0–12
B. Secondary descriptive categories	Knowledge ≥6/9 (study-defined)	158/485	32.6 (28.6–36.9)	—
	Attitude ≥8/11 (study-defined)	110/485	22.7 (19.2–26.6)	—
	Above-median safe-practice score (≥61.36)	253/485	52.2 (47.7–56.6)	—
	Any symptom	293/485	60.4 (56.0–64.7)	—

Continuous measures are presented as mean (SD), median (IQR) and observed range. Study-defined categories are provided for descriptive comparability only and are not externally validated clinical, regulatory or behavioral thresholds. Percentages are accompanied by Wilson 95% confidence intervals where shown.

**Figure 2:**
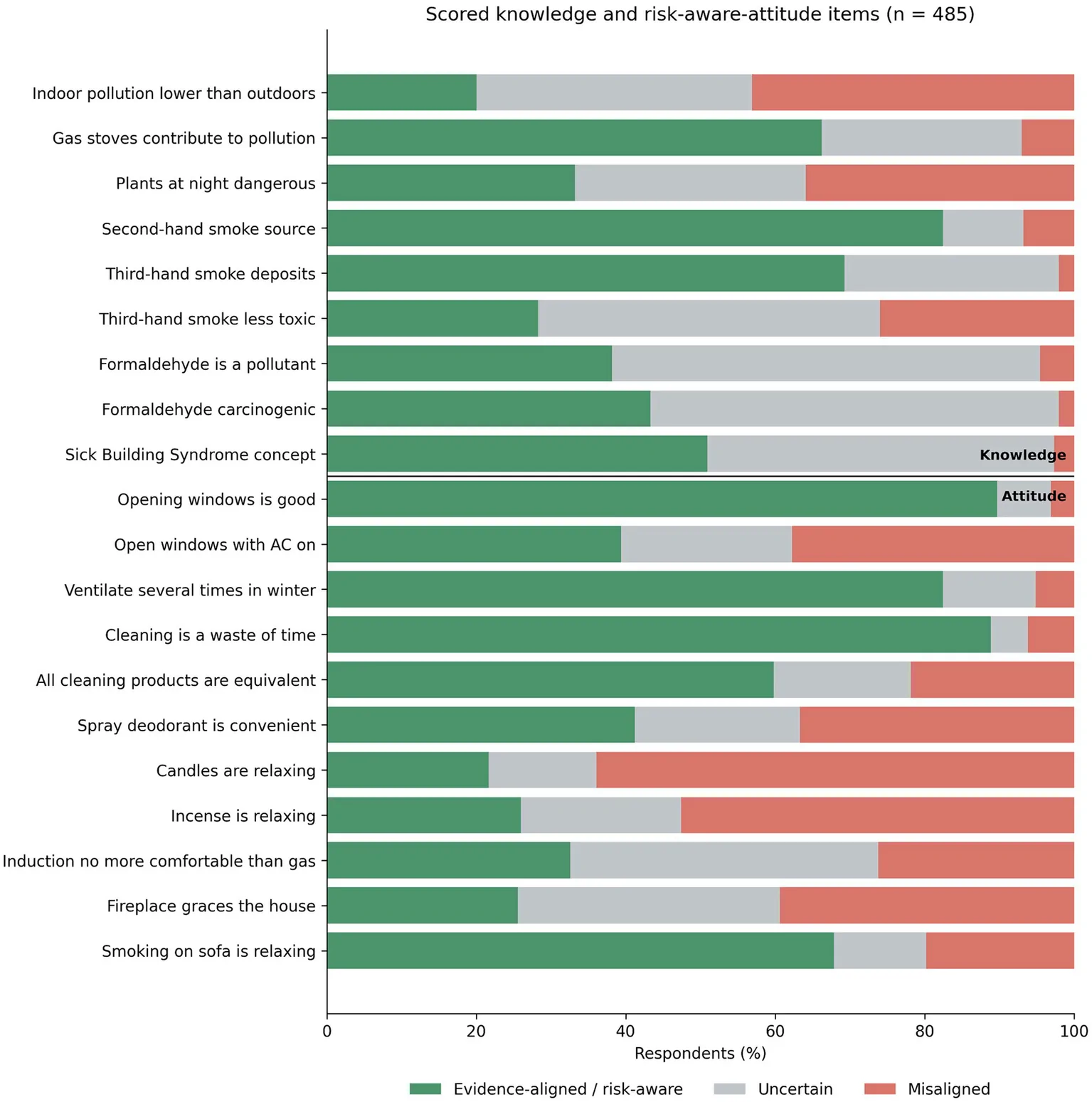
Item-level distributions for the nine scored knowledge items and eleven scored risk-aware-attitude items (*n* = 485). Each horizontal bar sums to 100%. Green indicates an evidence-aligned knowledge response or risk-aware attitude, gray indicates uncertainty, and red indicates a misaligned response. Uncertain responses are shown separately and are not interpreted as confidently incorrect beliefs. Three unscored knowledge statements and the unscored plant-aesthetic attitude item are reported in Supplementary Table S2.

**Figure 3:**
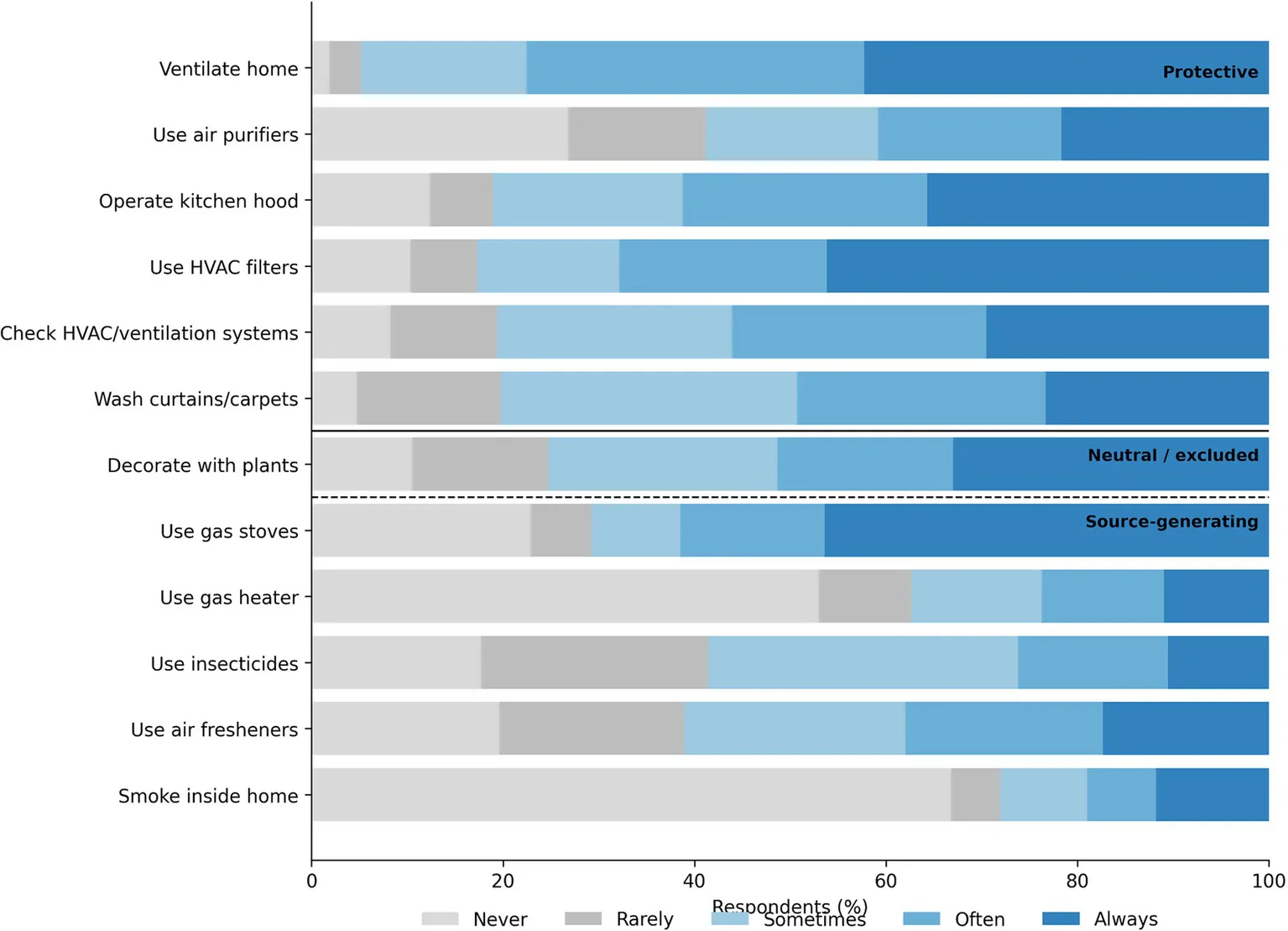
Frequency of reported household practices (*n* = 485). Stacked bars show Never, Rarely, Sometimes, Often and Always and sum to 100% for each item. Protective behaviors are grouped above the solid separator. Plant decoration is shown as neutral/excluded and was not included in the indices. Source-generating behaviors are grouped below the dotted separator. Classifications reflect the role of items in the study-specific indices and do not represent measured chemical exposure or equal health impact.

### 3.2 Knowledge and risk-aware attitude profiles

The mean confirmed-knowledge score was 4.32/9 (SD 2.08), with median 4.0 (IQR 3.0–6.0). The mean risk-aware-attitude score was 5.75/11 (SD 2.24), with median 6.0 (IQR 4.0–7.0) (Table 2). For secondary descriptive context only, 32.6% (158/485; 95% CI 28.6–36.9) scored ≥6/9 on knowledge and 22.7% (110/485; 95% CI 19.2–26.6) scored ≥8/11 on attitude. These cut-points are study-defined categories, not validated definitions of adequate knowledge or risk awareness.

Among the nine scored knowledge items (Figure 2), second-hand smoke from exhaled smoke had the highest evidence alignment (82.5%), followed by third-hand-smoke deposition (69.3%) and gas stoves as contributors to household pollution (66.2%). By contrast, 38.1% identified formaldehyde as a household pollutant and 43.3% selected the evidence-aligned response to the formaldehyde carcinogenicity item; uncertainty exceeded 50% for both. Uncertain responses received no point in the confirmed-knowledge score but were interpreted as lack of confirmed knowledge rather than affirmative false belief. Distributions for all 12 administered knowledge statements, including the three unscored items, are reported in Supplementary Table S2.

Only 28.2% selected the evidence-aligned disagreement with the statement that third-hand smoke is less toxic than second-hand smoke, while 45.8% were uncertain. Similarly, 33.2% rejected the statement that plants at night release substances dangerous to health. These item patterns illustrate uneven source-specific knowledge rather than a single latent knowledge trait and should be interpreted within the study-specific scoring framework.

Risk-aware responses were high for broad protective norms—89.7% agreed that opening windows is a good habit and 82.5% agreed that homes should be ventilated several times daily in winter, while risk-aware responses were lower for source-normalizing attitude items such as candles (21.6%), incense (26.0%), fireplaces (25.6%) and the induction-versus-gas item (32.6%). The complete distributions for all 12 attitude statements, including the unscored plant-aesthetic item, are reported in Supplementary Table S2.

Knowledge and risk-aware-attitude scores were positively correlated (Spearman ρ = 0.289, *p* < 0.001; FDR q < 0.001). In the primary adjusted continuous model, each one-standard-deviation increase in knowledge was associated with a 0.66-point higher attitude score (95% CI 0.47–0.86, *p* < 0.001). Relative to respondents aged 18–24 years, adjusted mean attitude scores were higher by 1.02 points at ages 25–34, 1.68 points at ages 35–44 and 1.63 points at ages ≥45; current smokers had a 0.55-point lower adjusted attitude score (Table 3). These are cross-sectional associations and do not establish a knowledge-to-attitude causal sequence.

**Table 3:** Selected primary multivariable model estimates.

Outcome	Predictor	Metric	Estimate (95% CI)	*p*	*n*
Risk-aware attitude	Knowledge alignment (per SD)	B	0.66 (0.47–0.86)	<0.001	481
	Age 25–34 (ref: 18–24)	B	1.02 (0.35–1.70)	0.003	481
	Age 35–44 (ref: 18–24)	B	1.68 (1.16–2.20)	<0.001	481
	Age > = 45 (ref: 18–24)	B	1.63 (1.09–2.18)	<0.001	481
	Current smoker (ref: non-smoker)	B	−0.55 (−0.95–-0.15)	0.007	481
Safe-practice index	Knowledge alignment (per SD)	B	1.72 (0.77–2.66)	<0.001	481
	Risk-aware attitude (per SD)	B	2.67 (1.63–3.71)	<0.001	481
	Age 35–44 (ref: 18–24)	B	3.23 (0.35–6.12)	0.028	481
	Age > = 45 (ref: 18–24)	B	4.76 (1.65–7.87)	0.003	481
	Current smoker (ref: non-smoker)	B	−4.31 (−6.57–-2.06)	<0.001	481
Any symptom	Protective practice (per SD)	OR	0.84 (0.68–1.03)	0.095	481
	Source-generating practice (per SD)	OR	1.11 (0.89–1.38)	0.352	481
	Male sex (ref: female)	OR	0.58 (0.37–0.90)	0.016	481
	Age 35–44 (ref: 18–24)	OR	0.43 (0.25–0.75)	0.003	481
	Age > = 45 (ref: 18–24)	OR	0.45 (0.25–0.81)	0.008	481
	High school or less (ref: bachelor)	OR	0.43 (0.23–0.79)	0.007	481
Symptom count	Protective practice (per SD)	IRR	0.90 (0.80–1.01)	0.082	481
	Source-generating practice (per SD)	IRR	1.16 (1.02–1.32)	0.020	481
	Age 35–44 (ref: 18–24)	IRR	0.51 (0.37–0.70)	<0.001	481
	Age > = 45 (ref: 18–24)	IRR	0.50 (0.35–0.72)	<0.001	481
	High school or less (ref: bachelor)	IRR	0.60 (0.42–0.87)	0.008	481

Continuous risk-aware attitude and safe-practice outcomes are reported as adjusted mean differences (B) from HC3 robust ordinary least-squares models; any symptom is reported as an odds ratio (OR); symptom count is reported as an incidence rate ratio (IRR) from negative-binomial regression. Continuous composite predictors are per one-standard-deviation increase. All models adjusted for gender, age group, education, smoking status and emirate. Reference categories were female, age 18–24 years, bachelor degree, non-smoker and Dubai. Complete model coefficients are provided in Supplementary Table S3.

### 3.3 Household practice patterns and cross-sectional associations

The protective-practice index averaged 65.21/100 (SD 18.53), the source-generating-practice index 42.04/100 (SD 21.79), and the formative safe-practice index 61.92/100 (SD 11.59) (Table 2). The median safe-practice index was 61.36; 52.2% of respondents were at or above that median, a secondary descriptive categorization that is not an external safety threshold.

Figure 3 shows that 77.5% of respondents reported ventilating the home often or always, 61.2% operated the kitchen hood often or always, 67.8% used HVAC filters often or always, and 56.1% periodically checked HVAC/ventilation systems often or always. Source-generating practices also occurred: gas-stove use was reported often or always by 61.4%, air-freshener use by 37.9%, insecticide use by 26.2%, gas-heater use by 23.7%, and indoor smoking at least rarely by 33.2%. These distributions describe reported behaviors and do not measure actual pollutant dose or exposure.

Exploratory correlations showed that knowledge was associated with protective practice (*ρ* = 0.219, FDR *q* < 0.001), lower source-generating practice (*ρ* = −0.115, *q* = 0.017) and higher safe-practice index (*ρ* = 0.247, *q* < 0.001). Risk-aware attitude was strongly inversely associated with source-generating practice (*ρ* = −0.496, *q* < 0.001) and positively associated with the combined safe-practice index (*ρ* = 0.385, *q* < 0.001), but was not positively associated with the protective component (*ρ* = −0.051, *q* = 0.302). Protective and source-generating indices were themselves positively correlated (*ρ* = 0.274, *q* < 0.001), underscoring behavioral coexistence and the non-unidimensional nature of the combined index (Supplementary Table S5).

In the primary adjusted continuous safe-practice model, each one-standard-deviation increase in risk-aware attitude was associated with a 2.67-point higher safe-practice index (95% CI 1.63–3.71, *p* < 0.001), while each one-standard-deviation increase in knowledge was associated with a 1.72-point higher index (95% CI 0.77–2.66, *p* < 0.001). Respondents aged 35–44 and ≥45 years had adjusted scores 3.23 and 4.76 points higher, respectively, than those aged 18–24 years; current smokers had a 4.31-point lower adjusted score. These cross-sectional differences are shown as selected estimates in Table 3 and Figure 4A; complete models appear in Supplementary Table S3.

**Figure 4:**
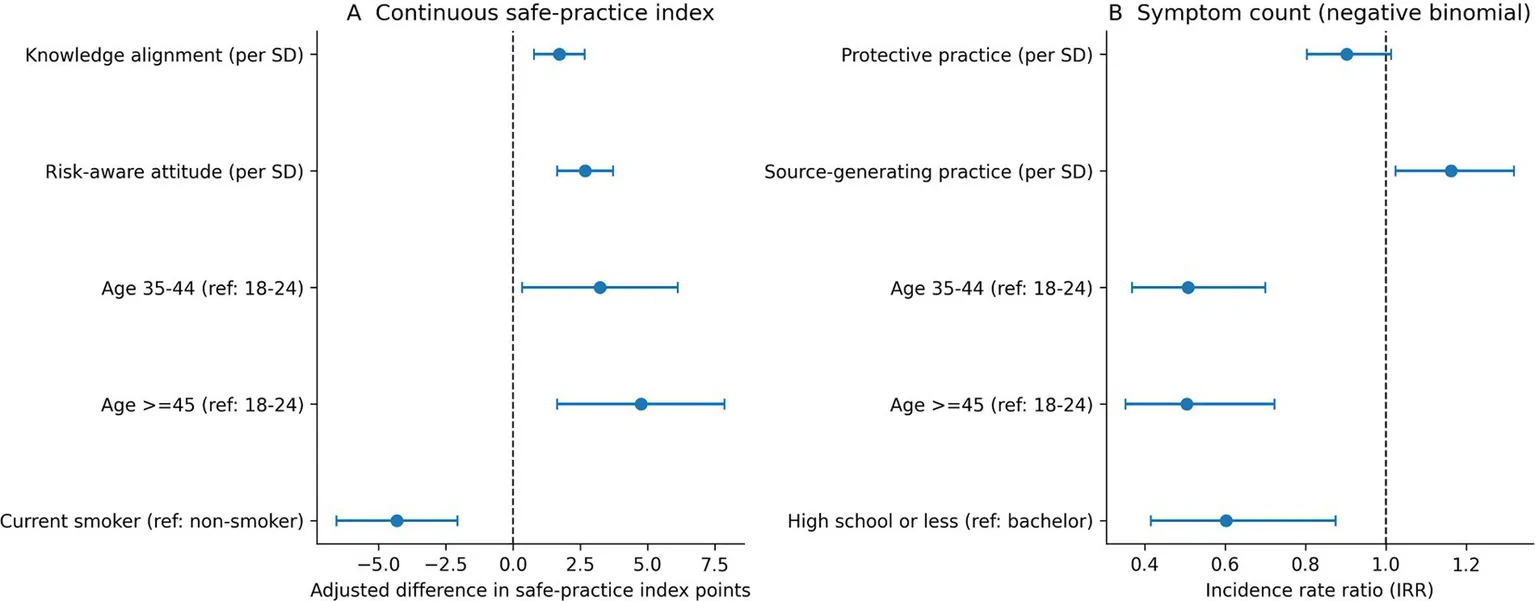
Selected adjusted associations (*n* = 481). Panel A shows adjusted differences (B coefficients) in the continuous safe-practice index from HC3 robust ordinary least-squares regression. Panel B shows incidence rate ratios (IRRs) from the negative-binomial symptom-count model. Continuous predictors are expressed per one-standard-deviation increase. Points are adjusted estimates and horizontal lines are 95% confidence intervals; the vertical dashed lines indicate the null (0 for B coefficients and 1 for IRRs). Models adjusted for gender, age group, education, smoking status and emirate. Reference categories were female, age 18–24 years, bachelor degree, non-smoker and Dubai. Only selected coefficients are displayed; complete model output is provided in Supplementary Table S3. Exact *p*-values are reported in the tables rather than added to the graphic.

Sensitivity analyses reinforced the need to interpret the formative practice construct by component. The alternative domain-balanced index was highly correlated with the original safe-practice index (r = 0.992) and retained positive adjusted associations with knowledge (*B* = 1.42 points per SD, 95% CI 0.47–2.36) and attitude (*B* = 3.29, 95% CI 2.26–4.32). However, component models showed heterogeneity: higher risk-aware attitude was associated principally with lower source-generating practice (*B* = −10.09 points per SD, 95% CI − 12.08 to −8.10) rather than uniformly higher protective-practice frequency. This pattern supports reporting the components separately and not treating the combined score as a unidimensional psychometric scale (Supplementary Table S4).

The coexistence of protective and source-generating behaviors means that households should not be labeled simply “safe” or “unsafe” from a single composite score. The findings instead identify distinct behavior domains that can be examined in future exposure studies. Source-specific communication hypotheses include cooking extraction, HVAC maintenance, smoke-free homes and reduced unnecessary fragranced emissions, but their effectiveness requires evaluation in representative and objectively monitored studies.

### 3.4 Nonspecific self-reported symptoms and adjusted associations

At least one listed nonspecific symptom was reported by 293 respondents (60.4%; 95% CI 56.0–64.7). Mean symptom count was 1.61 (SD 1.94), median 1.0 (IQR 0–3), and observed range 0–12. Headache was most frequently selected (41.6%), followed by dizziness/weakness (22.5%), stomach pain/cramping (17.1%), eye irritation-related symptoms (15.3%), chest pain (11.3%) and trouble breathing (11.3%) (Figure 5). These frequencies describe symptom reporting only and do not identify indoor chemicals as their cause.

**Figure 5:**
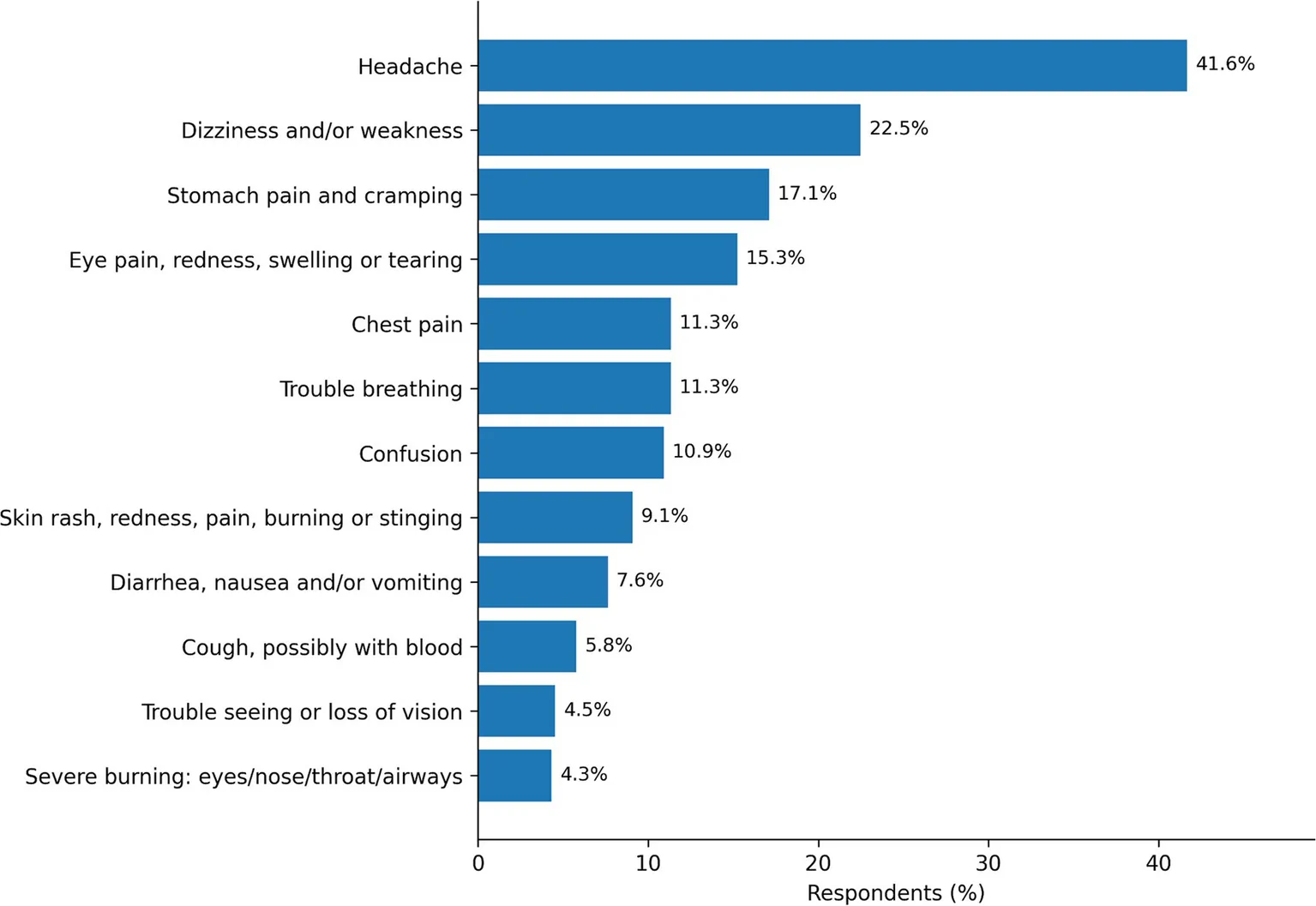
Prevalence of nonspecific self-reported symptoms among survey respondents (*n* = 485). Participants could select more than one symptom, so percentages do not sum to 100%. Symptoms were not clinically verified, were not linked temporally or spatially to measured indoor pollutants, and should not be interpreted as indicators of indoor chemical exposure.

In exploratory bivariate analysis, source-generating practice had a weak positive correlation with symptom count (*ρ* = 0.098, *p* = 0.031; FDR *q* = 0.042), while the safe-practice index was inversely correlated with symptom count (*ρ* = −0.153, *p* < 0.001; *q* = 0.001). Protective practice, knowledge and attitude were not materially correlated with symptom count after FDR consideration (Supplementary Table S5). These associations remain susceptible to unmeasured confounding and common-source self-report.

Adjusted results depended on symptom-outcome specification. In the logistic model for any symptom, source-generating practice was not statistically supported (OR 1.11 per SD, 95% CI 0.89–1.38, *p* = 0.352), and protective practice also had a confidence interval including the null (OR 0.84, 95% CI 0.68–1.03, *p* = 0.095). In the negative-binomial symptom-count model, source-generating practice was modestly associated with a higher reported symptom count (IRR 1.16 per SD, 95% CI 1.02–1.32, *p* = 0.020), whereas protective practice was not statistically supported (IRR 0.90, 95% CI 0.80–1.01, *p* = 0.082) (Table 3; Figure 4B). Robust Poisson and zero-inflated negative-binomial sensitivity models produced similar source-practice estimates (Supplementary Table S4).

In the negative-binomial model, respondents aged 35–44 years (IRR 0.51, 95% CI 0.37–0.70) and ≥45 years (IRR 0.50, 95% CI 0.35–0.72) reported lower symptom counts than the 18–24-year reference group. This finding should not be interpreted as biological protection. Age-related differences in reporting, sample composition, household routines, occupational exposure or other unmeasured factors may contribute and are considered in the limitations.

Taken together, the symptom analyses show a modest association between reported source-generating practices and the number of nonspecific symptoms, not evidence that household chemicals produced health effects. Pollutant concentrations, exposure timing, clinical diagnoses, symptom location and major alternative causes were not measured. The result is therefore hypothesis-generating and motivates future studies that pair time-resolved exposure measurements with validated health outcomes.

## 4 Discussion

### 4.1 Major findings

In this demographically skewed convenience sample of 485 adults, confirmed knowledge, risk-aware attitudes and household practices were heterogeneous. Formaldehyde, third-hand-smoke toxicity and several source-normalizing attitudes showed substantial uncertainty or misalignment, while protective behaviors coexisted with source-generating practices. Continuous adjusted models showed positive cross-sectional associations between knowledge and attitude and between both knowledge and attitude and the formative safe-practice index. These estimates characterize the analyzed respondents and should not be interpreted as national prevalence estimates or as evidence of a causal knowledge-attitude-practice pathway.

Risk-aware attitude showed a larger adjusted association with the combined safe-practice index than knowledge, but component analyses indicate that this association was driven primarily by lower source-generating behavior rather than uniformly greater protective behavior. This distinction is important: the formative index combines different actions and should not be interpreted as a single latent “safety” trait. The observed associations are compatible with behavioral theory in which risk appraisal, convenience, habits and social meaning accompany household choices, but the cross-sectional design cannot establish temporal ordering, mediation or causal mechanisms (12).

The symptom-count model showed a modest association between source-generating practice and a higher count of nonspecific self-reported symptoms, while the binary any-symptom association was not statistically supported. The result should therefore be described as greater reported symptom burden, not as a demonstrated indoor-chemical health effect. Stress, allergies, infections, chronic conditions, occupational exposures, outdoor pollution and other factors can produce the same symptoms, and common-source self-report and residual confounding remain substantial concerns.

Several reported symptoms overlap with building-related symptom profiles discussed in indoor-environment research (21, 22, 25), but the present study does not diagnose Sick Building Syndrome or attribute symptoms to an indoor source. Clinical assessment, time-location symptom diaries, objective pollutant measurements, ventilation-rate assessment and confirmation of symptom change with location were unavailable. The symptom findings therefore serve only as a hypothesis-generating basis for future exposure-linked research.

### 4.2 Novelty in relation to UAE and GCC evidence

The study contributes to a behavioral dimension that has been underdeveloped in the UAE indoor-air literature. The earlier UAE household study provided important evidence that incense use can relate to symptoms in homes and established that residential exposures in the UAE warrant health attention (16). Laboratory and source-characterization studies have further shown that incense and related scenting materials can emit pollutants of concern (17, 18). Reviews across GCC countries have called for better exposure assessment and more locally grounded evidence because high-temperature environments, dust, air-conditioning dependence and region-specific practices may affect indoor pollutant profiles (14, 15). The present analysis complements these studies by evaluating how residents understand and manage a range of indoor chemical hazards in everyday homes.

The contribution lies in examining knowledge, source-specific attitudes, household-practice domains and nonspecific symptom reporting in the same UAE sample. Recent UAE student studies have documented awareness deficits in specific academic populations (19, 20). The present convenience sample provides a different hypothesis-generating view, but the recruitment design and demographic concentration preclude claims of community or national representativeness.

The study also illustrates the importance of local adaptation and transparent scoring when using an international questionnaire. Montuori et al. (13) established a KAP framework for household chemical indoor pollution, while the UAE version introduced context-specific features such as incense, bilingual administration and a different practice-frequency scale. The absence of a formal UAE-specific cognitive pilot, back-translation and external validation means that observed score patterns should be interpreted as study-specific. Future validation should test semantic equivalence across languages and compare questionnaire scores with observed behavior, building characteristics and pollutant measurements.

### 4.3 Interpretation of specific knowledge gaps

The formaldehyde findings are particularly important. More than half of respondents were uncertain about whether formaldehyde is a household pollutant or a carcinogen. Formaldehyde is not a niche technical issue; it is a central indoor-air pollutant because emissions can originate from composite wood, insulation, adhesives, textiles, surface finishes and chemical reactions, and because exposure can occur chronically at low levels in homes (8, 9). Low recognition of formaldehyde suggests that risk communication should include invisible material emissions, not only smoke, odor or visible dust. Product labelling, low-emission procurement, ventilation after renovation and awareness of pressed-wood materials may be more actionable than abstract chemical lists.

Third-hand smoke provides an example of partial confirmed knowledge. Most respondents recognized that residues can remain indoors, but fewer selected the evidence-aligned response to the relative-toxicity item. Residues can persist on walls, furniture, curtains, carpets and dust and may undergo surface chemistry (10, 11). The survey result supports further source-specific education research, but it does not establish that smoking-related knowledge caused the observed practice differences.

The plant item illustrates a different type of misconception. Respondents were divided on whether plants at night release dangerous substances. The health concern implied by the item is not supported as a major indoor chemical-pollution priority; at the same time, claims that potted plants substantially purify indoor air are often exaggerated relative to real ventilation rates (28). This duality makes plants a useful educational example: they are not a substitute for source control and ventilation, but they also should not distract from higher-priority sources such as combustion, tobacco smoke, formaldehyde-emitting materials, pesticides and fragranced products.

These item-level findings identify candidate topics for future risk-communication research rather than population-level educational priorities. Formaldehyde, third-hand-smoke residues and source-normalizing beliefs around combustion and fragranced products can be evaluated in representative studies to determine which misunderstandings persist across emirates and demographic groups and which messages produce measurable behavioral or exposure changes.

### 4.4 Household practice coexistence and source control

A key professional interpretation is that households should not be classified as simply “safe” or “unsafe” on the basis of a single practice. The positive correlation between protective and source-generating practices indicates that some respondents performed more of both. This may reflect a general tendency toward active household management: the same households that ventilate, filter and clean may also use air fresheners, pesticides, scented candles or gas appliances. It may also reflect a compensation mindset in which ventilation or filtration is believed to neutralize source emissions. In practice, ventilation and filtration are important but may not fully remove gases, particles and secondary products, especially if sources are frequent or close to occupants.

Gas-stove use is a relevant example. Many respondents recognized gas stoves as a pollution source, yet gas-stove use was common. Kitchen-hood operation was also common, but the dataset did not measure hood capture efficiency, exhaust-to-outdoors configuration, maintenance status or use during every cooking event. Epidemiological evidence linking gas cooking and indoor nitrogen dioxide with asthma and wheeze indicates that risk reduction depends on source substitution, effective extraction and consistent use, not only general awareness (6). In UAE residences, where cooling and kitchen design may affect ventilation choices, future studies should measure hood performance, fuel type, cooking frequency and nitrogen dioxide concentrations together.

Fragranced products, candles and incense illustrate the challenge of sources that are intentionally introduced for comfort, identity or ambience. Respondents showed low risk-aware attitudes toward candles and incense, while air-freshener use was frequent. Peer-reviewed literature demonstrates that air fresheners and cleaning products can emit volatile chemicals and produce secondary pollutants indoors (4, 7, 29, 30). Incense smoke has been associated with particles and organic compounds in UAE-relevant studies, and daily incense use was linked with symptoms in the earlier UAE household study (16–18). Guidance should therefore avoid framing scenting practices as a matter of preference alone. Practical recommendations can include reducing frequency and duration, avoiding use in poorly ventilated rooms, keeping sources away from children and people with asthma, and selecting low-emission alternatives when possible.

Cleaning and pesticide practices require similar nuance. House cleaning was strongly valued by respondents, but disagreement with the statement that one cleaning product is as good as another was not universal, and insecticide use occurred often or always in more than one quarter of respondents. Cleaning can reduce dust reservoirs and allergens, but product selection and use patterns matter. International longitudinal evidence has linked household cleaning sprays with adult asthma, and mechanistic studies have shown that cleaning and fragranced products can contribute both primary and secondary pollutants (4, 7, 31). Indoor chemical-pollution guidance should therefore distinguish cleaning frequency from product chemistry, ventilation during use, mixing avoidance, dosage and storage.

### 4.5 Implications for future UAE research and risk communication

Because the sample was non-probability and demographically concentrated, the results should not be used to estimate UAE prevalence or set national policy priorities directly. They can generate candidate hypotheses and messages for evaluation in representative studies. Future work can test source-specific guidance concerning cooking extraction, HVAC maintenance, smoke-free homes and fragranced-product reduction while accounting for heat, humidity, dust and outdoor pollution when evaluating ventilation strategies.

Age was associated with both attitude and symptom reporting, but the study design does not support a mechanistic interpretation. The younger reference group differed substantially from older respondents in a convenience sample, and age-related reporting behavior, household roles, occupational exposures or other unmeasured factors may contribute. Future probability-based studies should determine whether age gradients persist before age-targeted interventions are prioritized.

Current smoking was associated with lower attitude and safe-practice scores in adjusted analyses. This sample-specific association is consistent with the relevance of second- and third-hand smoke to indoor-air communication, but it should not be generalized beyond the sample. Future intervention studies can assess whether residue-focused education changes household smoking practices and objectively measured exposure.

At a broader research level, the findings support measuring household behavior, product use and maintenance alongside pollutant concentrations in future UAE indoor-air studies. Representative sampling, objective exposure assessment and linkage to validated health outcomes are needed to determine whether the behavioral patterns observed here persist across emirates and whether changes in specific practices produce measurable exposure or health benefits.

### 4.6 Limitations

Several limitations should shape interpretation. The cross-sectional, self-reported design cannot establish temporality, mediation or causality. Recruitment used a non-probability convenience approach through university networks and in-person tablet-based collection; the sample was concentrated among females, Dubai residents and highly educated respondents, no sampling weights were available, and a population recruitment denominator could not be defined. Selection and nonresponse bias may therefore be substantial, and reported percentages are sample-specific. Four age values remained unresolved and were excluded only from age-adjusted models; no other model variables caused complete-case exclusions. Household pollutant concentrations, ventilation rates, room volumes, product ingredients, cooking frequency, HVAC performance, time spent at home and outdoor-air conditions were not measured.

The questionnaire was adapted from Montuori et al. (13) and administered in English and Arabic, but a separate UAE-specific cognitive pilot, formal translation/back-translation procedure and external validation were not documented. Some wording differed between language versions, including symptom framing, which may have influenced responses. The knowledge and attitude scores had modest internal consistency and used study-specific scoring rules; the ≥6/9 and ≥8/11 cut-points are not validated thresholds and are now secondary only. The formative safe-practice index had very low alpha and used equal item weighting despite behaviors having different potential exposure consequences; component analyses and an alternative domain-balanced index were therefore reported, but external validation remains necessary. Symptoms were nonspecific and could reflect infections, allergies, stress, chronic disease, occupational exposures, outdoor pollution or other causes; no clinical or time-location verification was available. Multiple analyses raise false-positive risk; inferential emphasis was placed on effect sizes and confidence intervals, item-level summaries were descriptive, and exploratory correlations were supplemented with Benjamini–Hochberg FDR q-values.

## 5 Conclusion

This cross-sectional questionnaire-based study of 485 adult UAE residents found heterogeneous confirmed knowledge, risk-aware attitudes and household practices within a demographically skewed convenience sample. In adjusted continuous models, higher knowledge was associated with higher risk-aware-attitude scores, and higher knowledge and risk-aware attitude were associated with a higher formative safe-practice index. Component analyses showed that the attitude association with the combined index was driven primarily by lower source-generating behavior, reinforcing that the index is formative and should not be interpreted as a unidimensional safety scale. These findings describe cross-sectional associations rather than a causal knowledge-attitude-practice pathway.

Source-generating household practices were modestly associated with a higher count of nonspecific self-reported symptoms, while the binary any-symptom association was not statistically supported. The study does not establish that indoor chemicals caused reported symptoms. Future research should use probability-based sampling across emirates, validated bilingual measures, objective time-resolved pollutant monitoring, building and HVAC characteristics, time-location information and validated health outcomes to determine population patterns and evaluate whether specific household interventions produce measurable exposure or health benefits.

## Data Availability

The raw data supporting the conclusions of this article will be made available by the authors, without undue reservation.
