# Supplementary material for: Knowledge, risk perception and household practices related to indoor chemical pollution in the United Arab Emirates
Source: Front Public Health. 2026 Oct 21;14:1926277. doi: 10.3389/fpubh.2026.1926277 (PMC13731276; doi:10.3389/fpubh.2026.1926277)
Supplement: Supplementary file 1 [file Supplementary_file_1.docx]

**Supplementary Materials**

*Knowledge, risk perception and household practices related to indoor chemical pollution in the United Arab Emirates*

Manuscript ID 1926277

This supplement provides questionnaire provenance and scoring, complete item-level distributions, full primary model outputs, sensitivity analyses, multiplicity-adjusted correlations, and participant-flow information requested during peer review.

# Supplementary Table S1. Questionnaire provenance, item role and scoring

The UAE questionnaire was adapted from Montuori et al. (2023). Knowledge and attitude items used Agree / Uncertain / Disagree response structures. Practice items used Never / Rarely / Sometimes / Often / Always, coded 0–4. The table documents every administered item and its analytic role. “Excluded from score” means the item remained in the questionnaire and descriptive supplement but did not contribute to the corresponding composite score.

## S1A. Knowledge items

| **ID** | **UAE English wording** | **Source** | **Adaptation** | **Analytic role** | **Evidence-aligned response / rationale** |
| --- | --- | --- | --- | --- | --- |
| K1 | The chemical pollution of the air in the household environment is less than that of outdoors. | Montuori K1 | Retained/adapted | Scored | Disagree |
| K2 | Gas stoves contribute to household pollution. | Montuori K2 | Retained/adapted | Scored | Agree |
| K3 | Plants at night release substances dangerous to health. | Montuori K3 | Retained/adapted | Scored | Disagree |
| K4 | Second-hand smoke comes from the smoke exhaled by a smoker. | Montuori K4 | Retained/adapted | Scored | Agree |
| K5 | Third-hand smoke derives from the toxic substances of the smoke deposited in the environment. | Montuori K5 | Retained/adapted | Scored | Agree |
| K6 | Third-hand smoke is less toxic than second-hand smoke. | Montuori K6 | Retained/adapted | Scored | Disagree |
| K7 | Inadequate ventilation causes more than 50% of the chemical pollution of the air in the domestic environment. | Montuori K7 | Retained/adapted | Excluded from score | Context-dependent numerical claim |
| K8 | The main household pollutant is carbon monoxide. | Montuori K8 | Retained/adapted | Excluded from score | Context-dependent “main pollutant” claim |
| K9 | Formaldehyde is one of the household chemical pollutants. | Montuori K9 | Retained/adapted | Scored | Agree |
| K10 | Formaldehyde is a certain carcinogen. | Montuori K10 | Retained/adapted | Scored | Agree |
| K11 | The Sick Building Syndrome is a condition in which the occupants of a building show a series of symptoms and pathologies without specific causes. | Montuori K11 | Retained/adapted | Scored | Agree |
| K12 | There are laws governing the pollution of domestic environments. | Montuori K12 | Retained/adapted | Excluded from score | Jurisdiction-specific claim |

## S1B. Attitude items

| **ID** | **UAE English wording** | **Source** | **Adaptation** | **Analytic role** | **Risk-aware response / rationale** |
| --- | --- | --- | --- | --- | --- |
| A1 | Opening windows is a good habit. | Montuori A1 | Retained/adapted | Scored | Agree |
| A2 | It is necessary to open the windows even with the air conditioner on. | Montuori A2 | Retained/adapted | Scored | Agree; study-specific risk orientation |
| A3 | In winter, it is still necessary to ventilate the house several times a day. | Montuori A3 | Retained/adapted | Scored | Agree |
| A4 | House cleaning is a waste of time. | Montuori A4 | Retained/adapted | Scored | Disagree |
| A5 | A cleaning product is as good as another. | Montuori A5 | Retained/adapted | Scored | Disagree |
| A6 | It is convenient to use spray deodorant. | Montuori A6 | Retained/adapted | Scored | Disagree |
| A7 | It is nice to have plants in the house. | Montuori A7 | Retained/adapted | Excluded from score | Aesthetic/neutral preference |
| A8 | Lightning candles at home is relaxing. | Montuori A8 | Retained/adapted | Scored | Disagree |
| A9 | Lightning incense at home is relaxing. | UAE-added | Context-specific addition | Scored | Disagree |
| A10 | Induction stoves are no more comfortable than the gas ones. | Montuori A9 | Retained/adapted | Scored | Disagree; favors non-combustion alternative |
| A11 | A fireplace graces the house. | Montuori A10 | Retained/adapted | Scored | Disagree |
| A12 | Smoking on the sofa is relaxing. | Montuori A11 | Retained/adapted | Scored | Disagree |

## S1C. Practice items

| **ID** | **UAE English wording** | **Source** | **Adaptation** | **Index role** | **Scoring** |
| --- | --- | --- | --- | --- | --- |
| P1 | Do you ventilate your home? | Montuori B1 | Retained/adapted | Protective | 0–4, higher = more frequent protective behavior |
| P2 | Do you use air purifiers? | Montuori B2 | Retained/adapted | Protective | 0–4 |
| P3 | Do you use gas stoves? | Montuori B3 | Retained/adapted | Source-generating | 0–4; reverse-coded in safe index |
| P4 | Do you operate the hood in the kitchen? | Montuori B4 | Retained/adapted | Protective | 0–4 |
| P5 | Do you use gas heater? | Montuori B5 | Simplified from gas and/or pellet heater | Source-generating | 0–4; reverse-coded in safe index |
| P6 | Do you use filters for heating/air conditioning systems? | Montuori B6 | Retained/adapted | Protective | 0–4 |
| P7 | Do you periodically check the heating, air conditioning, and ventilation systems? | Montuori B7 | Retained/adapted | Protective | 0–4 |
| P8 | Do you use insecticides at home? | Montuori B8 | Retained/adapted | Source-generating | 0–4; reverse-coded in safe index |
| P9 | Do you use air fresheners? | Montuori B9 | Retained/adapted | Source-generating | 0–4; reverse-coded in safe index |
| P10 | Do you wash curtains and carpets? | Montuori B10 | Retained/adapted | Protective | 0–4 |
| P11 | Do you decorate your home with plants? | Montuori B11 | Retained/adapted | Excluded from index | Descriptive only |
| P12 | Do you smoke in your home? | Montuori B12/B13 | Home-smoking items consolidated | Source-generating | 0–4; reverse-coded in safe index |

Symptom item: “Which symptom(s) do you regular have? (Select all that apply)” was study-specific and not derived from Montuori et al. It was analyzed only as nonspecific symptom reporting; “None of the above” was excluded from symptom count. The Arabic symptom prompt used poisoning-related wording, which is acknowledged as a potential language/framing limitation.

# Supplementary Table S2. Complete item-level response distributions

Counts and percentages are based on n = 485. Knowledge and attitude distributions include all administered items, not only those used in composite scores.

## S2A. Knowledge-item distributions

| **ID** | **Knowledge item** | **Agree** | **Uncertain** | **Disagree** |
| --- | --- | --- | --- | --- |
| K1 | The chemical pollution of the air in the household environment is less than that of outdoors. | 209 (43.1%) | 179 (36.9%) | 97 (20.0%) |
| K2 | Gas stoves contribute to household pollution. | 321 (66.2%) | 130 (26.8%) | 34 (7.0%) |
| K3 | Plants at night release substances dangerous to health. | 174 (35.9%) | 150 (30.9%) | 161 (33.2%) |
| K4 | Second-hand smoke comes from the smoke exhaled by a smoker. | 400 (82.5%) | 52 (10.7%) | 33 (6.8%) |
| K5 | Third-hand smoke derives from the toxic substances of the smoke deposited in the environment. | 336 (69.3%) | 139 (28.7%) | 10 (2.1%) |
| K6 | Third-hand smoke is less toxic than second-hand smoke. | 126 (26.0%) | 222 (45.8%) | 137 (28.2%) |
| K7 | Inadequate ventilation causes more than 50% of the chemical pollution of the air in the domestic environment. | 299 (61.6%) | 172 (35.5%) | 14 (2.9%) |
| K8 | The main household pollutant is carbon monoxide. | 219 (45.2%) | 214 (44.1%) | 52 (10.7%) |
| K9 | Formaldehyde is one of the household chemical pollutants. | 185 (38.1%) | 278 (57.3%) | 22 (4.5%) |
| K10 | Formaldehyde is a certain carcinogen. | 210 (43.3%) | 265 (54.6%) | 10 (2.1%) |
| K11 | The Sick Building Syndrome is a condition in which the occupants of a building show a series of symptoms and pathologies without specific causes. | 247 (50.9%) | 225 (46.4%) | 13 (2.7%) |
| K12 | There are laws governing the pollution of domestic environments. | 246 (50.7%) | 216 (44.5%) | 23 (4.7%) |

## S2B. Attitude-item distributions

| **ID** | **Attitude item** | **Agree** | **Uncertain** | **Disagree** |
| --- | --- | --- | --- | --- |
| A1 | Opening windows is a good habit. | 435 (89.7%) | 35 (7.2%) | 15 (3.1%) |
| A2 | It is necessary to open the windows even with the air conditioner on. | 191 (39.4%) | 111 (22.9%) | 183 (37.7%) |
| A3 | In winter, it is still necessary to ventilate the house several times a day. | 400 (82.5%) | 60 (12.4%) | 25 (5.2%) |
| A4 | House cleaning is a waste of time. | 30 (6.2%) | 24 (4.9%) | 431 (88.9%) |
| A5 | A cleaning product is as good as another. | 106 (21.9%) | 89 (18.4%) | 290 (59.8%) |
| A6 | It is convenient to use spray deodorant. | 178 (36.7%) | 107 (22.1%) | 200 (41.2%) |
| A7 | It is nice to have plants in the house. | 417 (86.0%) | 43 (8.9%) | 25 (5.2%) |
| A8 | Lightning candles at home is relaxing. | 310 (63.9%) | 70 (14.4%) | 105 (21.6%) |
| A9 | Lightning incense at home is relaxing. | 255 (52.6%) | 104 (21.4%) | 126 (26.0%) |
| A10 | Induction stoves are no more comfortable than the gas ones. | 127 (26.2%) | 200 (41.2%) | 158 (32.6%) |
| A11 | A fireplace graces the house. | 191 (39.4%) | 170 (35.1%) | 124 (25.6%) |
| A12 | Smoking on the sofa is relaxing. | 96 (19.8%) | 60 (12.4%) | 329 (67.8%) |

## S2C. Practice-item distributions

| **ID** | **Practice item** | **Never** | **Rarely** | **Sometimes** | **Often** | **Always** |
| --- | --- | --- | --- | --- | --- | --- |
| P1 | Do you ventilate your home? | 9 (1.9%) | 16 (3.3%) | 84 (17.3%) | 171 (35.3%) | 205 (42.3%) |
| P2 | Do you use air purifiers? | 130 (26.8%) | 70 (14.4%) | 87 (17.9%) | 93 (19.2%) | 105 (21.6%) |
| P3 | Do you use gas stoves? | 111 (22.9%) | 31 (6.4%) | 45 (9.3%) | 73 (15.1%) | 225 (46.4%) |
| P4 | Do you operate the hood in the kitchen? | 60 (12.4%) | 32 (6.6%) | 96 (19.8%) | 124 (25.6%) | 173 (35.7%) |
| P5 | Do you use gas heater? | 257 (53.0%) | 47 (9.7%) | 66 (13.6%) | 62 (12.8%) | 53 (10.9%) |
| P6 | Do you use filters for heating/air conditioning systems? | 50 (10.3%) | 34 (7.0%) | 72 (14.8%) | 105 (21.6%) | 224 (46.2%) |
| P7 | Do you periodically check the heating, air conditioning, and ventilation systems? | 40 (8.2%) | 54 (11.1%) | 119 (24.5%) | 129 (26.6%) | 143 (29.5%) |
| P8 | Do you use insecticides at home? | 86 (17.7%) | 115 (23.7%) | 157 (32.4%) | 76 (15.7%) | 51 (10.5%) |
| P9 | Do you use air fresheners? | 95 (19.6%) | 94 (19.4%) | 112 (23.1%) | 100 (20.6%) | 84 (17.3%) |
| P10 | Do you wash curtains and carpets? | 23 (4.7%) | 73 (15.1%) | 150 (30.9%) | 126 (26.0%) | 113 (23.3%) |
| P11 | Do you decorate your home with plants? | 51 (10.5%) | 69 (14.2%) | 116 (23.9%) | 89 (18.4%) | 160 (33.0%) |
| P12 | Do you smoke in your home? | 324 (66.8%) | 25 (5.2%) | 44 (9.1%) | 35 (7.2%) | 57 (11.8%) |

# Supplementary Table S3. Complete primary multivariable model outputs

All models used n = 481 complete cases. Reference categories: female; age 18–24 years; bachelor degree; non-smoker; Dubai. Continuous composite predictors are per one-standard-deviation increase. Intercepts are omitted from the table for readability.

| **Outcome** | **Predictor** | **Metric** | **Estimate** | **95% CI** | **p** |
| --- | --- | --- | --- | --- | --- |
| **Continuous risk-aware attitude** | Male sex (ref: female) | B | -0.105 | -0.538 to 0.328 | 0.634 |
|  | Age 25-34 (ref: 18-24) | B | 1.024 | 0.353 to 1.696 | 0.003 |
|  | Age 35-44 (ref: 18-24) | B | 1.677 | 1.156 to 2.197 | <0.001 |
|  | Age >=45 (ref: 18-24) | B | 1.632 | 1.087 to 2.178 | <0.001 |
|  | High school or less (ref: bachelor) | B | 0.013 | -0.643 to 0.669 | 0.968 |
|  | Postgraduate (ref: bachelor) | B | -0.112 | -0.601 to 0.377 | 0.653 |
|  | Current smoker (ref: non-smoker) | B | -0.551 | -0.953 to -0.150 | 0.007 |
|  | Abu Dhabi (ref: Dubai) | B | 0.083 | -0.386 to 0.552 | 0.730 |
|  | Other emirates (ref: Dubai) | B | -0.574 | -1.323 to 0.175 | 0.133 |
|  | Knowledge alignment (per SD) | B | 0.665 | 0.466 to 0.863 | <0.001 |
| **Continuous safe-practice index** | Male sex (ref: female) | B | -0.699 | -2.949 to 1.550 | 0.542 |
|  | Age 25-34 (ref: 18-24) | B | 0.767 | -2.268 to 3.802 | 0.621 |
|  | Age 35-44 (ref: 18-24) | B | 3.234 | 0.347 to 6.122 | 0.028 |
|  | Age >=45 (ref: 18-24) | B | 4.757 | 1.647 to 7.867 | 0.003 |
|  | High school or less (ref: bachelor) | B | -0.340 | -3.097 to 2.417 | 0.809 |
|  | Postgraduate (ref: bachelor) | B | 2.297 | -0.338 to 4.932 | 0.088 |
|  | Current smoker (ref: non-smoker) | B | -4.315 | -6.566 to -2.063 | <0.001 |
|  | Abu Dhabi (ref: Dubai) | B | -0.595 | -2.919 to 1.729 | 0.616 |
|  | Other emirates (ref: Dubai) | B | -3.082 | -6.000 to -0.164 | 0.038 |
|  | Knowledge alignment (per SD) | B | 1.718 | 0.774 to 2.663 | <0.001 |
|  | Risk-aware attitude (per SD) | B | 2.674 | 1.635 to 3.713 | <0.001 |
| **Any symptom** | Male sex (ref: female) | OR | 0.579 | 0.371 to 0.904 | 0.016 |
|  | Age 25-34 (ref: 18-24) | OR | 0.675 | 0.344 to 1.326 | 0.254 |
|  | Age 35-44 (ref: 18-24) | OR | 0.428 | 0.245 to 0.748 | 0.003 |
|  | Age >=45 (ref: 18-24) | OR | 0.447 | 0.247 to 0.811 | 0.008 |
|  | High school or less (ref: bachelor) | OR | 0.430 | 0.234 to 0.791 | 0.007 |
|  | Postgraduate (ref: bachelor) | OR | 1.352 | 0.819 to 2.232 | 0.239 |
|  | Current smoker (ref: non-smoker) | OR | 1.118 | 0.706 to 1.771 | 0.635 |
|  | Abu Dhabi (ref: Dubai) | OR | 1.043 | 0.654 to 1.662 | 0.861 |
|  | Other emirates (ref: Dubai) | OR | 1.070 | 0.529 to 2.165 | 0.850 |
|  | Protective practice (per SD) | OR | 0.839 | 0.682 to 1.031 | 0.095 |
|  | Source-generating practice (per SD) | OR | 1.110 | 0.891 to 1.382 | 0.352 |
| **Symptom count** | Male sex (ref: female) | IRR | 0.827 | 0.638 to 1.072 | 0.151 |
|  | Age 25-34 (ref: 18-24) | IRR | 0.787 | 0.542 to 1.145 | 0.211 |
|  | Age 35-44 (ref: 18-24) | IRR | 0.508 | 0.369 to 0.700 | <0.001 |
|  | Age >=45 (ref: 18-24) | IRR | 0.505 | 0.353 to 0.723 | <0.001 |
|  | High school or less (ref: bachelor) | IRR | 0.603 | 0.416 to 0.875 | 0.008 |
|  | Postgraduate (ref: bachelor) | IRR | 1.162 | 0.858 to 1.575 | 0.331 |
|  | Current smoker (ref: non-smoker) | IRR | 1.087 | 0.831 to 1.422 | 0.543 |
|  | Abu Dhabi (ref: Dubai) | IRR | 1.233 | 0.949 to 1.603 | 0.117 |
|  | Other emirates (ref: Dubai) | IRR | 0.980 | 0.666 to 1.441 | 0.917 |
|  | Protective practice (per SD) | IRR | 0.902 | 0.803 to 1.013 | 0.082 |
|  | Source-generating practice (per SD) | IRR | 1.162 | 1.024 to 1.319 | 0.020 |

# Supplementary Table S4. Sensitivity and alternative-construction analyses

## S4A. Study-defined knowledge and attitude thresholds

| **Analysis** | **Category / threshold** | **n (%)** | **Adjusted knowledge association** | **95% CI; p** |
| --- | --- | --- | --- | --- |
| Knowledge prevalence only | Knowledge ≥5/9 | 223 (46.0%) | Not modeled as primary binary outcome | — |
| Knowledge prevalence only | Knowledge ≥6/9 | 158 (32.6%) | Not modeled as primary binary outcome | — |
| Knowledge prevalence only | Knowledge ≥7/9 | 72 (14.8%) | Not modeled as primary binary outcome | — |
| Secondary logistic sensitivity | Attitude ≥7/11 | 177 (36.5%) | OR 1.71 | 1.37–2.12; p=<0.001 |
| Secondary logistic sensitivity | Attitude ≥8/11 | 110 (22.7%) | OR 1.56 | 1.22–1.98; p=<0.001 |
| Secondary logistic sensitivity | Attitude ≥9/11 | 53 (10.9%) | OR 1.49 | 1.09–2.03; p=0.013 |

## S4B. Practice-index construction and component models

| **Outcome / sensitivity** | **Predictor** | **Metric** | **Estimate** | **95% CI** | **p** |
| --- | --- | --- | --- | --- | --- |
| Above-median safe-practice score | Knowledge alignment (per SD) | OR | 1.22 | 0.98 to 1.51 | 0.078 |
|  | Risk-aware attitude (per SD) | OR | 1.97 | 1.54 to 2.51 | <0.001 |
| Domain-balanced safe-practice index | Knowledge alignment (per SD) | B | 1.42 | 0.47 to 2.36 | 0.003 |
|  | Risk-aware attitude (per SD) | B | 3.29 | 2.26 to 4.32 | <0.001 |
| Protective-practice index | Knowledge alignment (per SD) | B | 4.73 | 3.04 to 6.43 | <0.001 |
|  | Risk-aware attitude (per SD) | B | -3.51 | -5.49 to -1.52 | <0.001 |
| Source-generating-practice index | Knowledge alignment (per SD) | B | 1.90 | 0.09 to 3.72 | 0.040 |
|  | Risk-aware attitude (per SD) | B | -10.09 | -12.08 to -8.10 | <0.001 |

The original equal-item safe-practice index and domain-balanced alternative had Pearson r = 0.992.

## S4C. Symptom-count model specification

| **Model** | **Source-generating IRR (95% CI)** | **p** | **Protective IRR (95% CI)** | **p** | **Fit / diagnostic** |
| --- | --- | --- | --- | --- | --- |
| Negative binomial (primary) | 1.16 (1.02–1.32) | 0.020 | 0.90 (0.80–1.01) | 0.082 | AIC 1654.8 |
| Robust Poisson (HC3) | 1.15 (1.02–1.30) | 0.027 | 0.92 (0.81–1.04) | 0.162 | Sensitivity to count-model variance assumption |
| Zero-inflated negative binomial | 1.17 (1.04–1.31) | 0.008 | 0.92 (0.83–1.02) | 0.103 | AIC 1644.1; intercept-only inflation |

Complete-case symptom count: mean 1.61, variance 3.80. Ordinary Poisson overdispersion diagnostics: Pearson χ²/df = 2.12 and deviance/df = 2.15. The negative-binomial dispersion estimate was α = 0.79 (95% CI 0.57–1.01). Observed zero proportion was 39.9% versus 37.5% predicted by the negative-binomial model. The zero-inflated model had lower AIC but did not materially change the source-practice estimate; the simpler negative-binomial model was retained as primary because a separate structural-zero process was not prespecified.

# Supplementary Table S5. Exploratory Spearman correlations with Benjamini–Hochberg FDR adjustment

FDR adjustment was applied across all 15 pairwise Spearman correlations among the six continuous/ordinal-derived measures. Item-level response distributions were descriptive and were not subjected to inferential multiple testing.

| **Variable 1** | **Variable 2** | **Spearman ρ** | **p** | **BH-FDR q** |
| --- | --- | --- | --- | --- |
| Knowledge | Risk-aware attitude | 0.289 | <0.001 | <0.001 |
| Knowledge | Protective practice | 0.219 | <0.001 | <0.001 |
| Knowledge | Source-generating practice | -0.115 | 0.011 | 0.017 |
| Knowledge | Safe-practice index | 0.247 | <0.001 | <0.001 |
| Knowledge | Symptom count | 0.016 | 0.727 | 0.727 |
| Risk-aware attitude | Protective practice | -0.051 | 0.261 | 0.302 |
| Risk-aware attitude | Source-generating practice | -0.496 | <0.001 | <0.001 |
| Risk-aware attitude | Safe-practice index | 0.385 | <0.001 | <0.001 |
| Risk-aware attitude | Symptom count | -0.048 | 0.295 | 0.316 |
| Protective practice | Source-generating practice | 0.274 | <0.001 | <0.001 |
| Protective practice | Safe-practice index | 0.567 | <0.001 | <0.001 |
| Protective practice | Symptom count | -0.052 | 0.254 | 0.302 |
| Source-generating practice | Safe-practice index | -0.575 | <0.001 | <0.001 |
| Source-generating practice | Symptom count | 0.098 | 0.031 | 0.042 |
| Safe-practice index | Symptom count | -0.153 | <0.001 | 0.001 |

# Supplementary Figure S1. Participant and analytic-sample flow

Figure S1. All 485 retained respondents were adults after verification of apparent age-entry errors. Four unresolved age values were missing only for age-adjusted models, giving n = 481. No other model variables caused complete-case exclusions.

# Appendix S1. Administered bilingual questionnaire wording

The following reproduces the English and Arabic item wording supplied for the study questionnaire. Minor spelling or grammatical features are retained because the purpose is to document the administered wording rather than retrospectively edit it. The English dataset export did not store the Arabic response-label strings; therefore, response structures are documented separately: Agree / Uncertain / Disagree for knowledge and attitude items, and Never / Rarely / Sometimes / Often / Always for practice items.

## Appendix S1A. Knowledge statements

| **ID** | **English wording** | **Arabic wording** |
| --- | --- | --- |
| K1 | The chemical pollution of the air in the household environment is less than that of outdoors. | التلوث الكيميائي للهواء داخل المنزل أقل من خارج المنزل |
| K2 | Gas stoves contribute to household pollution. | تسهم الأفران-المواقد الغازية في تلوث المنزل. |
| K3 | Plants at night release substances dangerous to health. | تُنتج النباتات ليلاً مواد خطيرة على الصحة. |
| K4 | Second-hand smoke comes from the smoke exhaled by a smoker. | التدخين غير المباشر (المستوى الثاني) يحدث بسبب التدخين المباشر للمدخن الرئيسي (المستوى الأول) |
| K5 | Third-hand smoke derives from the toxic substances of the smoke deposited in the environment. | التدخين غير المباشر (المستوى الثالث) يحدث بسبب المواد السامة للدخان المتواجدة في البيئة. |
| K6 | Third-hand smoke is less toxic than second-hand smoke. | التدخين غير المباشر (المستوى الثالث) أقل خطورة من التدخين غير المباشر (المستوى الثاني). |
| K7 | Inadequate ventilation causes more than 50% of the chemical pollution of the air in the domestic environment. | تتسبب ضعف التهوئة في أكثر من 50٪ من التلوث الكيميائي للهواء داخل المنزل |
| K8 | The main household pollutant is carbon monoxide. | الملوث الرئيسي في المنازل هو أول أكسيد الكربون. |
| K9 | Formaldehyde is one of the household chemical pollutants. | مادة "الفورم الديهايد" هي واحدة من الملوثات الكيميائية المنزلية |
| K10 | Formaldehyde is a certain carcinogen. | مادة "الفورم الديهايد" قد تسبب السرطان |
| K11 | The Sick Building Syndrome is a condition in which the occupants of a building show a series of symptoms and pathologies without specific causes. | متلازمة المنزل المرضي هي حالة يعاني منها سكان المنزل بسلسلة من الأعراض والاعتلالات دون أسباب محددة. |
| K12 | There are laws governing the pollution of domestic environments. | هناك قوانين تنظم تلوث البيئات المنزلية |

## Appendix S1B. Attitude statements

| **ID** | **English wording** | **Arabic wording** |
| --- | --- | --- |
| A1 | Opening windows is a good habit. | فتح النوافذ عادة جيدة |
| A2 | It is necessary to open the windows even with the air conditioner on. | عادة فتح النوافذ مع تشغيل مكيف الهواء ضرروية |
| A3 | In winter, it is still necessary to ventilate the house several times a day. | في الشتاء، من الضروري تهوئة المنزل عدة مرات في اليوم |
| A4 | House cleaning is a waste of time. | تنظيف المنزل مضيعة للوقت |
| A5 | A cleaning product is as good as another. | كل منتجات التنظيف جيدة دون فرق |
| A6 | It is convenient to use spray deodorant. | إستخدام مزيل العرق الرذاذ عادة صحية |
| A7 | It is nice to have plants in the house. | وجود النباتات في المنزل عادة جميلة |
| A8 | Lightning candles at home is relaxing. | إشعال الشموع في المنزل مريح |
| A9 | Lightning incense at home is relaxing. | إشعال البخور في المنزل مريح |
| A10 | Induction stoves are no more comfortable than the gas ones. | أفران الكهرباء ليست أكثر راحة من أفران الغاز |
| A11 | A fireplace graces the house. | المدفأة تزين المنزل |
| A12 | Smoking on the sofa is relaxing. | التدخين على الأريكة مريح |

## Appendix S1C. Practice questions

| **ID** | **English wording** | **Arabic wording** |
| --- | --- | --- |
| P1 | Do you ventilate your home? | هل تقوم بتهوئة منزلك؟ |
| P2 | Do you use air purifiers? | هل تستخدم أجهزة تنقية الهواء؟ |
| P3 | Do you use gas stoves? | هل تستخدم أفران الغاز؟ |
| P4 | Do you operate the hood in the kitchen? | هل تقوم بتشغّيل الشفاط في المطبخ؟ |
| P5 | Do you use gas heater? | هل تستخدم سخان الغاز؟ |
| P6 | Do you use filters for heating/air conditioning systems? | هل تستخدم فلاتر لأنظمة التدفئة/تكييف الهواء؟ |
| P7 | Do you periodically check the heating, air conditioning, and ventilation systems? | هل تفحص بشكل دوري أنظمة التدفئة والتكييف والتهوية؟ |
| P8 | Do you use insecticides at home? | هل تستخدم المبيدات الحشرية في المنزل؟ |
| P9 | Do you use air fresheners? | هل تستخدم معطرات الهواء؟ |
| P10 | Do you wash curtains and carpets? | هل تغسل الستائر والسجاد؟ |
| P11 | Do you decorate your home with plants? | هل تزين منزلك ؟بالنباتات |
| P12 | Do you smoke in your home? | هل تدخن في منزلك؟ |

## Appendix S1D. Symptom item

| **Field** | **English** | **Arabic** |
| --- | --- | --- |
| **Symptom prompt** | Which symptom(s) do you regular have? (Select all that apply) | الأعراض الناتجة عن التسمم (اختر كل ما ينطبق) |

Symptom response options were multi-select and included: headache; dizziness and/or weakness; stomach pain and cramping; eye pain/redness/swelling/tearing; trouble breathing; chest pain; confusion; skin rash/redness/pain/burning/stinging; diarrhea/nausea/vomiting; cough possibly with blood; trouble seeing/loss of vision; severe burning in eyes/nose/throat/airways; and “None of the above.”
